# NRF2 and Primary Cilia: An Emerging Partnership

**DOI:** 10.3390/antiox9060475

**Published:** 2020-06-02

**Authors:** Ana Martin-Hurtado, Isabel Lastres-Becker, Antonio Cuadrado, Francesc R. Garcia-Gonzalo

**Affiliations:** 1 Instituto de Investigaciones Biomédicas Alberto Sols (IIBM), UAM-CSIC, 28029 Madrid, Spain; amartinh@cnio.es (A.M.-H.); ilbecker@iib.uam.es (I.L.-B.); antonio.cuadrado@uam.es (A.C.); 2 Departamento de Bioquímica, Facultad de Medicina, Universidad Autónoma de Madrid (UAM), 28029 Madrid, Spain; 3 Instituto de Investigación del Hospital Universitario de La Paz (IdiPAZ), 28047 Madrid, Spain; 4 Centro de Investigación Biomédica en Red sobre Enfermedades Neurodegenerativas (CIBERNED), ISCIII, 28013 Madrid, Spain

**Keywords:** NRF2, primary cilia, hedgehog signaling, ciliogenesis, ciliopathy, cancer, autophagy, mTOR, non-small cell lung cancer (NSCLC)

## Abstract

When not dividing, many cell types target their centrosome to the plasma membrane, where it nucleates assembly of a primary cilium, an antenna-like signaling structure consisting of nine concentric microtubule pairs surrounded by membrane. Primary cilia play important pathophysiological roles in many tissues, their dysfunction being associated with cancer and ciliopathies, a diverse group of congenital human diseases. Several recent studies have unveiled functional connections between primary cilia and NRF2 (nuclear factor erythroid 2-related factor 2), the master transcription factor orchestrating cytoprotective responses to oxidative and other cellular stresses. These NRF2-cilia relationships are reciprocal: primary cilia, by promoting autophagy, downregulate NRF2 activity. In turn, NRF2 transcriptionally regulates genes involved in ciliogenesis and Hedgehog (Hh) signaling, a cilia-dependent pathway with major roles in embryogenesis, stem cell function and tumorigenesis. Nevertheless, while we found that NRF2 stimulates ciliogenesis and Hh signaling, a more recent study reported that NRF2 negatively affects these processes. Herein, we review the available evidence linking NRF2 to primary cilia, suggest possible explanations to reconcile seemingly contradictory data, and discuss what the emerging interplay between primary cilia and NRF2 may mean for human health and disease.

## 1. Primary Cilia as Cellular Antennae

**Figure 1:**
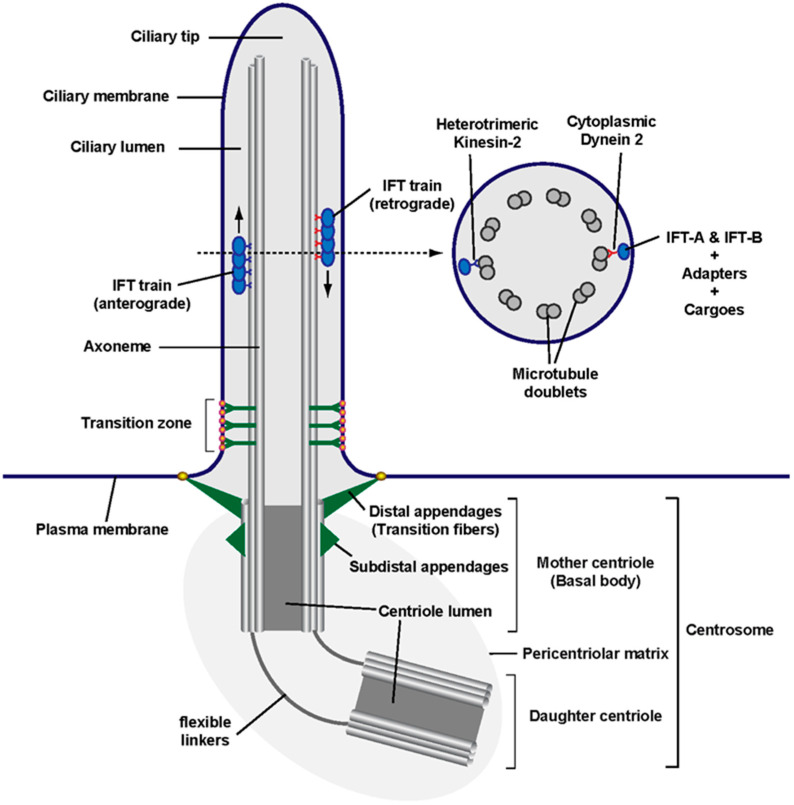
Anatomy of a primary cilium: Schematic of a primary cilium and its parts, including the centrosome at its base, from whose mother centriole (known as basal body in the context of a cilium) emanates the ciliary axoneme (i.e., its microtubule shaft). The region separating the ciliary compartment from the rest of the cell contains two main structures: the mother centriole’s distal appendages (known as transition fibers in a ciliary context) and the transition zone, with its distinctive Y-shaped linkers ending in bead-like membrane particles known as the ciliary necklace. Along the axoneme travel intraflagellar transport (IFT) trains, which move towards the ciliary tip (anterograde) or the ciliary base (retrograde). As shown in the cross section (right), the primary cilium axoneme consists of nine microtubule doublets but lacks structures found in motile cilia and flagella (such as a central microtubule pair, radial spokes and axonemal dynein arms). As indicated, IFT trains are multiprotein assemblies consisting of microtubule motors and IFT complexes IFT-A and IFT-B. These protein complexes can associate with cargo directly (as is the case for α/β-Tubulin dimers) or indirectly through adapters such as the BBSome, which connects IFT complexes to membrane cargoes. The main anterograde motor is heterotrimeric kinesin-2 (with its KIF3A, KIF3B and KAP subunits), whereas cytoplasmic dynein-2 is the retrograde motor [1,6,9].

Primary cilia are non-motile microtubule-based structures that protrude from the plasma membrane and act as cellular antennae, transducing mechanical, optical or chemical signals in a cell type-specific manner [1,2,3,4,5]. The ciliary endoskeleton, or axoneme, consists of nine concentrically arranged microtubule pairs that emanate from the basal body, the membrane-docked mother centriole of the cellular centrosome (Figure 1). Because of this centrosomal specialization, ciliated cells typically do not divide without first disassembling their primary cilium. Accordingly, primary cilia are normally found in quiescent cells and are absent from rapidly dividing cell populations such as cancer cells or early embryo blastomers [6].

Primary cilia are present in most mammalian cell types, the main exception being the hematopoietic lineage, where centrosomes play alternative, highly specialized functions, as in the immunological synapse [7]. In ciliated cell types, ciliogenesis occurs shortly after mitotic exit, upon entry into G0/G1 [6]. An essential role in ciliogenesis is played by intraflagellar transport (IFT) trains, whose key components include two multiprotein complexes, IFT-A and IFT-B, and their associated microtubule motors: kinesin-2 for anterograde motion (from cilium base to tip) and cytoplasmic dynein 2 for retrograde (back to base) transport (Figure 1) [8,9]. Like elevators moving up and down a growing skyscraper, IFT trains carry building blocks, like tubulin dimers, to the ciliary tip, where the cilium is extended. Eventually, a dynamic equilibrium is reached where ciliary tip extension is offset by its disassembly, leading to a constant ciliary length, which can vary considerably between cell types and under different conditions [8]. For instance, increased gene expression of IFT train components and ciliary building blocks can increase ciliary length, and vice versa [10,11].

**Figure 2:**
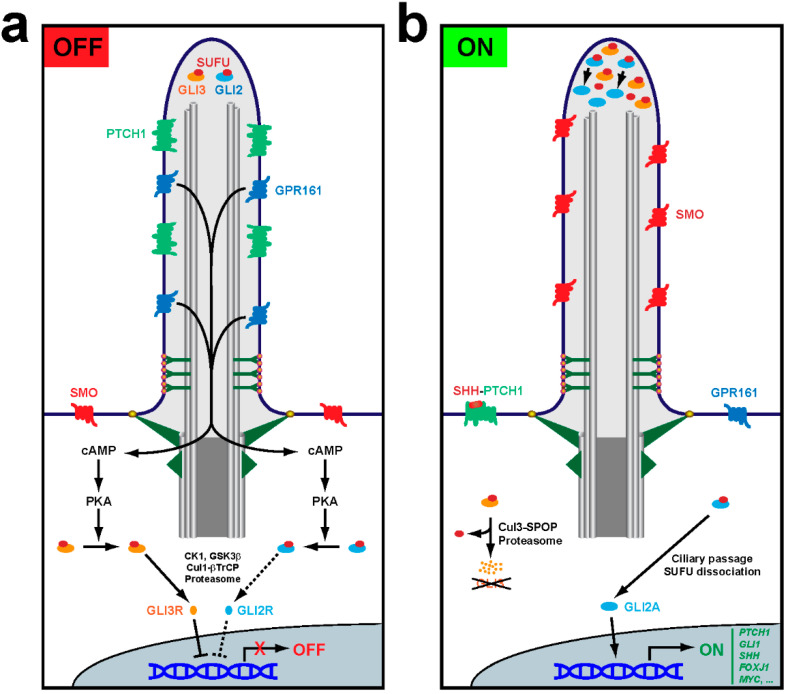
Primary cilia control Hedgehog pathway output. (**a**) In the OFF mode, Hh ligands are absent and their receptor PTCH1 accumulates inside cilia, keeping SMO outside. GPR161, a constitutively active G_s_ protein-coupled receptor, promotes ciliary production of cAMP, which activates protein kinase A (PKA) at the ciliary base. PKA then phosphorylates GLI2 and GLI3, the transcription factors mediating Hh pathway output. Both factors interact with their repressor SUFU, with which they travel to and from the ciliary tip. Such travel is required for PKA-dependent regulation of GLI2 and GLI3. Phosphorylation by PKA primes GLI2 and GLI3 for phosphorylation by casein kinase 1 (CK1) and glycogen synthase kinase 3β (GSK3β). For GLI3, this leads to ubiquitination by the Cul1-βTrCP (cullin1-β-transducin repeats-containing protein) E3 ubiquitin ligase and subsequent proteasome-dependent partial proteolytic cleavage to release SUFU and form GLI3R, a transcriptional repressor. GLI2 can also be processed into GLI2R, but this is inefficient because GLI2, unlike GLI3, lacks a processing-determinant domain (PDD). Thus, most phosphorylated GLI2 remains SUFU-bound and inactive. When cilia are absent or dysfunctional, unstimulated cells cannot form GLI3R, and thus its target genes are derepressed. This is the molecular mechanism underlying certain manifestations of ciliopathies, like polydactyly. (**b**) In the ON mode, Hh ligands like SHH bind PTCH1, removing it from cilia and enabling ciliary accumulation and activation of SMO. This leads to GPR161 ciliary exit, which stops cAMP synthesis and prevents PKA activation. In the absence of PKA phosphorylation, GLI3 is fully degraded by the proteasome via Cul3-SPOP (Cullin3-Speckle-type POZ protein)-dependent ubiquitination, whereas GLI2 is no longer prevented from ridding itself of SUFU at the ciliary tip and turning into the GLI2A transcriptional activator. Both GLI2 and GLI3 strongly accumulate at the ciliary tip under these conditions. In the nucleus, lack of GLI3R and presence of GLI2A leads to activation of Hh target genes such as *PTCH1* and *GLI1*, which exert negative and positive pathway feedback, respectively. Other Hh target genes are cell type-specific, such as *SHH* and *FOXJ1*, which are induced in the floor plate of the embryonic neural tube by notochord-derived SHH ligand [15,16].

Once formed, primary cilia carry out their signaling functions. For that, it is essential that all appropriate receptors and signal transducers accumulate at the ciliary membrane prior to signal reception [1,2,3,4,5]. Moreover, upon pathway activation, signal transduction sometimes requires protein movements into or out of cilia, as occurs in Hedgehog (Hh) signaling, a ciliary signaling pathway that is essential for embryonic development and stem cell function, and whose constitutive activation causes cancer (Figure 2) [12,13,14,15].

In response to Hh ligands, including Sonic Hedgehog (SHH), Indian Hedgehog (IHH) or Desert Hedgehog (DHH), the transmembrane receptor Patched (PTCH1) disappears from cilia, allowing ciliary accumulation of Smoothened (SMO), a G protein-coupled receptor (GPCR). Activation of SMO causes the ciliary departure of another GPCR, GPR161, which in turn leads to the glioma-associated (GLI) transcription factors accumulating at the ciliary tip. There, they dissociate from their repressor Suppressor of Fused (SUFU) and undergo modifications that, after exiting cilia, will affect their transcriptional output in the cell nucleus (Figure 2) [12,16]. All this dynamic trafficking in fully formed cilia also requires IFT trains, which connect to their membrane cargoes via adapters like the BBSome, the Bardet–Biedl syndrome (BBS) protein complex (Figure 1, Table 1) [3].

Besides Hh signaling, primary cilia are implicated in a wide variety of signaling pathways [1,2,3]. Many other GPCRs, from rhodopsin in the retina to serotonin receptors in the brain, signal through cilia, not only through G proteins but also through the mammalian target of rapamycin (mTOR) and other pathways [17,18]. Other proteins localized in cilia include receptor tyrosine kinases like platelet-derived growth factor receptor (PDGFR), insulin-like growth factor receptor (IGFR) and the neurotrophin receptor p75/NTR, as well as mechanosensitive channels like the calcium-permeating polycystins [1,2,19]. Mutations in the latter cause polycystic kidney disease (PKD), the most prevalent of a diverse group of congenital human diseases known as ciliopathies, whose manifestations may include retinal degeneration, cystic kidneys, brain malformations, obesity and polydactyly, among others [1,20,21]. In addition to ciliopathies, primary cilia are also implicated in cancer [6,13,14]. Cancer progression often correlates with loss of primary cilia, which can function as tumor suppressors [6,14]. However, primary cilia are also known to promote tumorigenesis, as occurs in medulloblastomas and basal cell carcinomas driven by constitutively active ciliary Hh signaling (Table 1) [13,14]. Other diseases in which primary cilia play increasingly recognized roles include diabetes, obesity, muscular dystrophy, and psychiatric and neurodegenerative diseases [18,22,23,24,25,26,27,28,29,30]. 

In many of these diseases, a prominent role is played by redox, proteostatic and other kinds of cellular stress [31,32,33,34,35,36,37,38]. Consistently, primary cilia and Hh signaling can respond to redox and other stress signals, and their function may also protect cells from such stresses [39,40,41,42,43,44,45,46,47,48,49,50,51]. In this context, recent findings connecting primary cilia and Hh signaling to NRF2 (nuclear factor erythroid 2-related factor 2) are of special relevance. These findings are reviewed in the next sections. 

## 2. NRF2 and Its Links to Primary Cilia

NRF2, encoded by the *NFE2L2* gene, is a basic region-leucine zipper transcription factor belonging to the cap-n-collar (CNC) family and best known as a master regulator of cellular antioxidant and detoxification responses [52,53,54]. Under non-stressed conditions, NRF2 binds the E3 ubiquitin ligase adapter Kelch-like ECH-associated protein 1 (KEAP1) in the cytoplasm, leading to NRF2 degradation via the ubiquitin-proteasome system. By acting on critical KEAP1 cysteines, oxidative stress interferes with KEAP1-mediated NRF2 degradation, allowing translocation of NRF2 into the nucleus, where it activates hundreds of target genes whose promoters contain NRF2-binding antioxidant response elements (AREs). NRF2 targets include genes for many antioxidant and detoxifying enzymes that help cells cope with stress. Among such enzymes are those promoting synthesis of NADPH, an essential cofactor in antioxidant reactions, and reduced glutathione (GSH), a key cellular redox buffer. Other NRF2-regulated factors include redoxin family proteins, key sensors of reactive oxygen species (ROS) production at specific intracellular compartments. Other protective NRF2 effects include processes such as preserving mitochondrial DNA integrity and respiratory chain function, producing the nonpolar antioxidant bilirubin, or preventing quinones from depleting cellular GSH pools [54]. Some NRF2 target genes mentioned below include *GCLC* (encoding the catalytic subunit of glutathione cysteine ligase, a GSH synthetic enzyme), *HMOX1* (encoding heme oxygenase 1, involved in bilirubin synthesis) and *NQO1* (encoding NAD(P)H: quinone oxidoreductase 1, which detoxifies quinones) [52,53,54].

**Figure 3:**
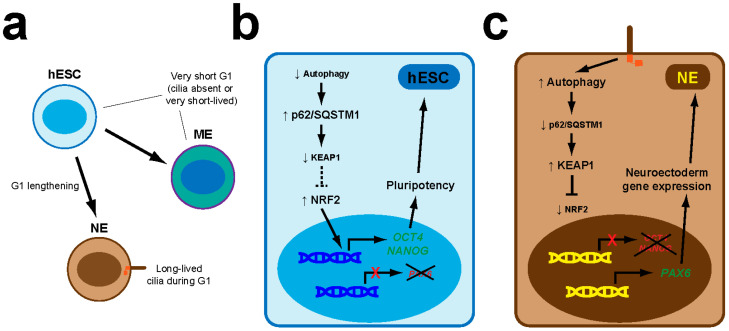
The primary cilia-autophagy-NRF2 (PAN) axis controls neuroectoderm specification in hESCs. (**a**) hESCs divide rapidly and their G1 cell cycle phase is too short for primary cilia to appear (if they do, they are quickly disassembled before the next cell division). As hESC offspring begin the process of neuroectoderm (NE) lineage specification, G1 lengthens, allowing primary cilia to form for longer periods. Such G1 lengthening does not occur in the early phases of mesendoderm (ME) specification. (**b**) In hESCs, autophagy levels are low, allowing p62/SQSTM1 to accumulate and promote KEAP1 degradation. As a result, NRF2 activity is high and promotes expression of pluripotency genes like *OCT4* and *NANOG*, which block induction of NE markers like *PAX6*. These circuits are not altered during early ME specification. (**c**) In the NE lineage, the PAN axis is set in motion: cilia induce higher autophagy, leading to lower p62/SQSTM1 protein levels, KEAP1 upregulation and NRF2 downregulation. Consequently, *OCT4* and *NANOG* expression is switched off, enabling upregulation of *PAX6* and other NE genes [57].

NRF2 targets also include genes for regulators of autophagy, another stress-coping mechanism [55,56]. However, other targets are not so clearly connected to stress, if at all. For instance, NRF2 promotes expression of the pluripotency genes *OCT4* and *NANOG* in human embryonic stem cells (hESCs) [57]. For these cells to differentiate into neuroectoderm, NRF2-dependent expression of these genes must be repressed via the primary cilia-autophagy-NRF2 (PAN) axis, the first reported connection between primary cilia and NRF2 (Figure 3) [57]. More recently, we found the PAN axis is also operative in fibroblasts, even though pluripotency and neuroectoderm differentiation are not at stake in these cells [58].

Furthermore, as we also showed, primary cilia-NRF2 connections are bidirectional, as NRF2 affects ciliogenesis and Hh signaling, both of which it regulates transcriptionally [58,59]. Interestingly, though, while we found a positive effect of NRF2 on cilia and Hh signaling [58,59], a very recent study found a negative connection instead [58,59]. In the remainder of this paper, we will review the reciprocal functional interactions between primary cilia and NRF2, discussing their possible pathophysiological meanings and proposing possible explanations where the evidence appears contradictory.

## 3. Primary Cilia Downregulation of NRF2 via Autophagy

Autophagy is a cellular process of self-degradation in which autophagy-related (ATG) proteins orchestrate engulfment of intracellular components in double membrane vacuoles known as autophagosomes, whose fusion with lysosomes gives rise to autophagolysosomes where engulfed components are digested, releasing their building blocks, which cells can use to generate energy or renew their ageing machinery. Energy generation helps cells survive stresses such as starvation, whereas self-renewal helps cells stay fit for longer, explaining why vigorous basal autophagy extends organismal lifespan. Too much or too little autophagy, however, is associated with cancer, neurodegeneration and other diseases [60,61,62,63].

The bidirectional interplay between ciliogenesis and autophagy has been reviewed elsewhere [64,65]. Briefly, autophagy controls ciliogenesis by targeting ciliogenic regulators for degradation. These regulators can be positive, like IFT20, or negative, like the orofaciodigital syndrome protein OFD1, perhaps explaining why autophagy enhances or represses ciliogenesis in different contexts [66,67,68,69]. Conversely, autophagy flux is reduced by depletion of key ciliogenic proteins, like kinesin-2 subunit KIF3A or IFT-B subunits IFT20 and IFT88 [57,67,69]. Moreover, Hh pathway activation in fibroblasts stimulates autophagy, an effect requiring the above IFT proteins [67,70]. However, Hh signaling cannot be the only mechanism whereby cilia promote autophagy, as cilia still upregulate autophagy in the absence of Hh pathway activation. Consistently, several ATG proteins localize to the ciliary base and two of them, VPS34 and ATG16L, translocate to the ciliary base in an IFT-dependent manner upon autophagy induction [67].

As with cilia, autophagy’s connection to NRF2 also goes both ways. On the one hand, NRF2 stimulates autophagy gene expression, raising autophagic flux [55,56]. On the other hand, KEAP1-dependent NRF2 levels are controlled by p62/sequestosome 1 (SQSTM1), an autophagy cargo receptor that traffics ubiquitinated proteins to autophagosomes [71,72,73,74]. Besides recruiting cargo, p62 is also an autophagy substrate. Hence, autophagy inhibition causes p62 to accumulate, leading to its phosphorylation by mammalian target of rapamycin complex 1 (mTORC1) [71]. Phosphorylated p62 then targets KEAP1 for autophagic degradation, thus activating NRF2, which proceeds to activate its target genes, including *p62/SQSTM1* itself, leading to a positive feedback loop whose malfunction is implicated in cancer and neurodegeneration [71,72,73,74,75,76]. Conversely, increased autophagy reduces p62 levels and is associated with increased NRF2 inhibition, as seen with the PAN axis (Figure 3) [57]. 

The hESC cell cycle is too fast for cilia to emerge. However, as hESC differentiation to neuroectoderm begins, G1 lengthening allows primary cilia formation during G1, which sets the PAN axis in motion, leading in succession to a rise in autophagy flux, reduced p62 levels, NRF2 transcriptional activity decrease, *OCT4* and *NANOG* downregulation, and expression of *PAX6* and other neuroectoderm markers (Figure 3) [57].

**Figure 4:**
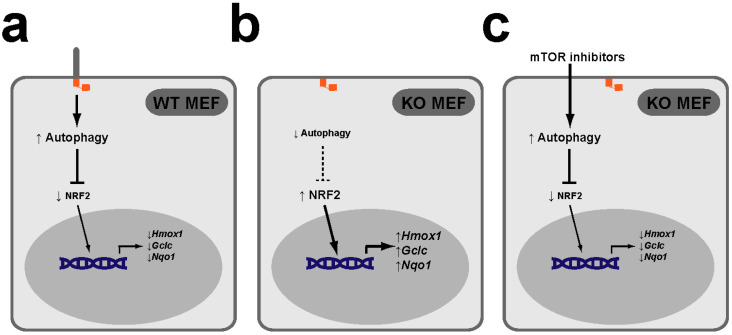
The PAN axis also functions in mouse embryonic fibroblasts (MEFs). (**a**) In wild type (WT) MEFs, the PAN axis functions to keep NRF2 transcriptional activity low, as reflected in low expression of NRF2 targets *Hmox1*, *Gclc* and *Nqo1*. (**b**) In MEFs KO for essential ciliogenic genes, such as *Kif3a* or *Ift88*, lack of cilia leads to lower levels of autophagy and higher NRF2 target gene expression levels. (**c**) Treatment of KO MEFs with mTOR inhibitors rapamycin or torin-1 increases their autophagy levels and restores NRF2 target expression to normal (low) levels [58].

The PAN axis is not restricted to early embryogenesis, however. As we recently showed, it also operates in mouse embryonic fibroblasts (MEFs) derived from E11.5 embryos (Figure 4) [58]. While knockdown of KIF3A, IFT88 or IFT20 in hESCs blocks their PAN axis-dependent neuroectodermal differentiation, ciliogenesis-defective *Kif3a*^−/−^ or *Ift88*^−/−^ MEFs show a clear upregulation of the NRF2 transcriptional targets *Gclc*, *Hmox1* and *Nqo1*, indicating that primary cilia repress NRF2 in both contexts [57,58]. Moreover, the abnormally high NRF2 activity in cilia-null MEFs can be rescued with mTOR inhibitors, which raise autophagic flux in these cells (as in many others), as assessed by the chloroquine-induced rise in (LC3B-II)/(LC3B-I) ratio, a measure of how fast MAP1LC3B (microtubule-associated protein 1 light chain 3B), an autophagy receptor and substrate, is degraded in autophagolysosomes [58]. Together with our finding that Hh signaling is not involved in cilia-dependent NRF2 regulation in MEFs, these data indicate that, in both MEFs and hESCs, primary cilia downregulate NRF2 activity by stimulating autophagy (Figure 3 and Figure 4), consistent with the above-mentioned studies where the cilia-autophagy and autophagy-NRF2 connections were separately analyzed [55,56,57,58,64,65,73,74].

Nevertheless, some questions remain. For instance, how does increased autophagy reduce NRF2 activity in hESCs and MEFs? As mentioned above, higher autophagy levels should lead to lower p62 levels, which in turn would lead to higher KEAP1 and lower NRF2 protein levels. Consistently, p62 and KEAP1 proteins were indeed decreased and increased, respectively, upon neuroectoderm differentiation of hESCs, in which NRF2 protein was not examined [57]. In our study, we did not examine p62 or KEAP1 in cilia-null MEFs, but we did look at NRF2 protein levels, which, to our surprise, were not appreciably altered [58]. If confirmed, this would suggest that the increased NRF2 activity in *Kif3a*^−/−^ MEFs may not be mediated by the p62-KEAP1 pathway. Known mechanisms affecting NRF2 activity independently of its levels include changes in the levels of its binding partners, such as the small musculoaponeurotic fibrosarcoma (sMAF) factors, or changes in NRF2 subcellular localization, as occurs in Hutchinson-Gilford progeria syndrome, where progerin, a lamin A mutant, retains NRF2 at the nuclear periphery [53]. Further studies are warranted to elucidate the details of how cilia affect NRF2 activity.

Another outstanding question concerns the pathophysiological roles of the PAN axis in cell types other than hESCs. This is still a mystery, but a few attractive hypotheses can be proposed. First, the PAN axis may control cell fate choices in other stem cell types, as it does in hESCs (Figure 3). In contrast, differentiated cells might use PAN signaling as a sensor of ciliary damage or stress. According to this hypothesis, intact cilia would promote high levels of basal autophagy and low levels of Nrf2 activity. This status quo would be disrupted by perturbations in ciliary structure or function, leading to NRF2 activity upregulation, which would help cells fix the problem, for instance by inducing ciliary gene expression. This could help explain, for example, some features of chronic obstructive pulmonary disease (COPD), where NRF2 cytoprotection is enlisted downstream of cilia damage [77]. Another interesting model is the renal epithelium, where urine flow signals through primary cilia to repress mTOR, induce autophagy and thereby reduce cell volume [78,79]. If the PAN axis is functional in these cells, one would predict NRF2 activity to be kept at low levels by this pathway, and to rise upon pathway disruption, as may occur in PKD [20,80]. Although this remains speculative, some evidence links NRF2 to kidney cystogenesis [81]. Future studies are needed to clarify which cell types have a functional PAN circuit, and what they use it for.

## 4. NRF2 Upregulation of Primary Cilia and Hh Signaling

Given the above-described bidirectional relationships between primary cilia and autophagy, on the one hand, and between NRF2 and autophagy, on the other, we hypothesized the PAN axis could function backwards, with NRF2 affecting cilia via autophagy (or NAP axis) [55,56,57,58,64,65,73,74]. To test this, we examined ciliary function in NRF2-null MEFs. Since Hh signaling is highly reliant on ciliary structure, composition and dynamics, we used it as a readout of ciliary function [12,58,82,83,84,85]. We therefore measured the induction of two Hh target genes, *Gli1* and *Ptch1*, by the SMO agonist SAG, a Hh pathway activator [58,86]. Twenty-four hours after SAG treatment of wild type (WT) MEFs, both target genes were induced 10- to 15-fold relative to vehicle-treated MEFs. In contrast, SAG-induced *Gli1* and *Ptch1* mRNA levels in NRF2-null MEFs were about 5-fold lower compared to wild type, a highly significant difference. Although baseline *Gli1* and *Ptch1* levels displayed a downward trend in NRF2-null cells, this difference was not significant. These data came from multiple independent experiments in immortalized MEFs; they were confirmed by GLI1 protein immunoblot and replicated in the original primary MEFs [58]. Thus, NRF2 is a positive regulator of SAG-induced Hh signaling in MEFs.

Nevertheless, because not all events in Hh signaling are cilia-dependent, we also examined cilia in NRF2-null MEFs. After twenty-four hours of serum starvation, about 70% of WT MEFs displayed primary cilia, whose average length was ≈3.5 µm. In NRF2-null MEFs these numbers were 40% and ≈3.0 µm, both differences being very significant. Reduced ciliation was also confirmed in primary MEFs, confirming it is not an immortalization artefact. Furthermore, we also found ciliogenesis defects in vivo, in astrocytes of the mouse hippocampus, a brain region whose function strongly relies on both NRF2 and primary cilia [58,87,88,89,90,91,92,93,94,95,96,97,98]. The ciliogenic defects in NRF2-null hippocampus were even stronger than in MEFs, with cilia numbers reduced about 5-fold. Hence, NRF2 is a positive regulator of ciliogenesis, explaining, at least partly, why it also promotes Hh signaling [58].

We then examined the mechanisms whereby NRF2 promotes ciliogenesis and Hh signaling. Our first hypothesis was that lower autophagy in NRF2-null cells leads to accumulation of ciliogenic repressors like OFD1, causing the observed phenotypes [58,68]. If so, experimentally increasing autophagy in NRF2-null cells should rescue their defects. In contrast to this hypothesis, autophagy upregulation by mTOR inhibition did not improve Hh responsiveness in NRF2-null cells [58]. Alternatively, NRF2 mutant cells could have abnormally high levels of ciliophagy, or ciliary autophagy, a histone deacetylase 6 (HDAC6)-dependent process [77,99]. However, this is probably not the case either, because the HDAC6 inhibitor tubastatin A does not affect Hh signaling in NRF2-null MEFs [58]. Another possibility is that reduced ciliogenesis is a byproduct of increased redox stress. If so, N-acetylcysteine, which helps cells replenish their glutathione stores, should have improved Hh responses in NRF2-null cells, but this was not the case either [58,100]. Likewise, PKA inhibition by H89 had no effect, which argues against overactivation of PKA, a Hh pathway repressor, as the responsible mechanism (Figure 2) [58].

As a next step, we examined whether NRF2 promotes ciliogenic gene expression. The mRNA levels of all five ciliogenic genes studied (*Ift74*, *Ift88*, *Ift172*, *Ift140* and *Dync2h1*) were all significantly downregulated by about 30–50% in NRF2-null MEFs, likely explaining their ciliogenic defects [58]. A parsimonious explanation of these data would be that NRF2 promotes expression of one or more ciliogenic transcription factors, which would then upregulate IFT and other ciliogenic genes. Since the above genes are all known targets of the regulatory factor X (RFX) family transcription factors, we evaluated them next [10,101]. However, well-established ciliogenic RFX factors (*Rfx1-4*) were not downregulated in NRF2-null MEFs [10,58,101]. Instead, reductions were seen for *Rfx5* and *Rfx7*, but it is unclear whether this is related to ciliogenesis, as these factors are better known as immunity regulators [10,101,102,103]. Alternatively, NRF2 may act on ciliogenic genes not through other transcription factors, but directly. To assess this possibility, we performed a bioinformatic search for ARE sequences in the promoters of the above ciliogenic genes in human. Interestingly, *IFT74*, *IFT88*, *IFT172* and *IFT140* all have high-scoring putative ARE sequences, suggesting that NRF2 may directly upregulate them [58]. However, the functionality of these AREs and their impact on ciliogenesis remain untested.

Overall, the evidence indicates that the likely reason why NRF2-null cells have fewer and shorter cilia is that ciliogenic gene upregulation is NRF2-dependent. Nevertheless, the moderate ciliogenic defects of these cells only partially explain the marked reduction in Hh responsiveness, suggesting that NRF2 has additional effects on Hh signaling. Indeed, the expression of genes affecting Hh signaling but not ciliogenesis is also reduced in NRF2-null MEFs. All such genes we analyzed, namely *Smo*, *Gpr161*, *Gli2*, *Gli3, Sufu* and *Ift27*, showed reduced expression levels. Moreover, we identified high-confidence ARE sequences in the promoters of *Gpr161*, *Gli2*, *Gli3* and *Ift27*, with the putative AREs in *Gli2* and *Gli3* being perfect matches with the ARE consensus sequence [58]. We also found candidate AREs in *Ptch1*, the Hh receptor and target gene that has recently been confirmed as a *bona fide* direct NRF2 target [58,59]. Further evidence that *Gpr161* and *Ift27* (and the ciliogenic *Ift74*) are NRF2 targets comes from their promoters being identified in a ChIP-seq experiment aiming to identify NRF2-binding sites [104]. Thus, NRF2 affects Hh responsiveness both via ciliogenesis and via Hh pathway gene expression, which it controls directly, at least in some cases.

The NRF2-dependent Hh pathway components include both positive (*Smo*, *Gli2*, *Ift27*) and negative (*Gpr161*, *Gli3, Sufu*, *Ptch1*) regulators of the Hh pathway, such that the net effects of their downregulation on Hh responsiveness are not immediately obvious [12,58]. For example, one consideration is that *Ptch1* is not only a Hh/GLI target gene but also, as shown recently, an NRF2 target gene; thus, the reduction we observed in SAG-treated NRF2-null cells may be explained, at least partly, by lack of direct NRF2 activation [58,59]. This may also be the reason why we found, as mentioned above, a downward trend in unstimulated *Ptch1* levels in NRF2-null cells. The fact that this reduction was not significant may be due to a low signal-to-noise ratio when dealing with the very low baseline *Ptch1* expression levels [58]. A related question is whether the Hh target *Gli1* is also a direct NRF2 target gene. Although we found no putative AREs in the *Gli1* promoter, such AREs may not have reached our cutoff score, or they may lie outside the analyzed region. In fact, the pattern of *Gli1* expression in untreated versus SAG-treated NRF2-null MEFs was similar to that of *Ptch1*, including a downward trend in baseline levels. Thus, it is possible that the reduced Hh responsiveness we observed in NRF2-null cells might be explained, at least partly, by direct NRF2 effects on Hh target genes [58]. 

However, there is so far no direct evidence indicating that *Gli1* is an NRF2 target gene. Moreover, the observed NRF2-dependent changes in *Ptch1* expression are weak compared to those in other NRF2 target genes, and therefore lack of direct NRF2 activation may explain only a small fraction of the strong reduction in SAG-induced *Ptch1* levels observed in NRF2-null cells [58,59]. Consistently, we detected no increase in *Gli1* or *Ptch1* mRNAs in WT MEFs upon NRF2 stabilization with dimethyl fumarate, an electrophile activator of NRF2 [58,105,106,107]. This suggests that newly accumulated NRF2 molecules do not stimulate endogenous *Gli1* or *Ptch1*, or do so only weakly.

Aside from any direct effects of NRF2 on *Gli1* and *Ptch1* gene expression, our data likely reflect reduced Hh signal transduction in NRF2-null cells, as expected from their cilia defects and altered levels of Hh pathway components. Among these, the effects of GLI2 and GLI3 are expected to predominate, because: (i) they function downstream of PTCH1, SMO, GPR161 and SUFU; (ii) the aforementioned lack of rescue by H89 indicates that Hh signaling defects in NRF2-null cells are downstream of PKA, which directly phosphorylates GLI2 and GLI3; and (iii) GLI2 and GLI3 directly regulate Hh target genes (levels of the other GLI transcription factor, GLI1, are exceedingly low until Hh signaling activates its gene expression) (Figure 2) [12]. For all these reasons, we focused our analysis on GLI2 and GLI3.

Upon Hh pathway activation in wild type cells, GLI2 and GLI3 accumulate at the ciliary tip, which is necessary for SUFU dissociation and GLI2 processing into its activator form, GLI2A. In NRF2-null MEFs, not only are overall GLI2 and GLI3 protein levels reduced, but SAG-induced ciliary tip accumulation of both GLI2 and GLI3 is also decreased, indicating lower levels of Hh signal transduction in these cells. Since IFT27-null cells display similar defects in GLI2 and GLI3, it is possible that NRF2 effects on GLI2 and GLI3 are partly mediated by reduced IFT27 levels [108]. On the other hand, these effects on GLI2 and GLI3 should be epistatic over any effects caused by reduced levels of Hh pathway repressors like SUFU, GPR161 or PTCH1, all of which act upstream of IFT27, GLI2 and GLI3 [12,108,109].

**Figure 5:**
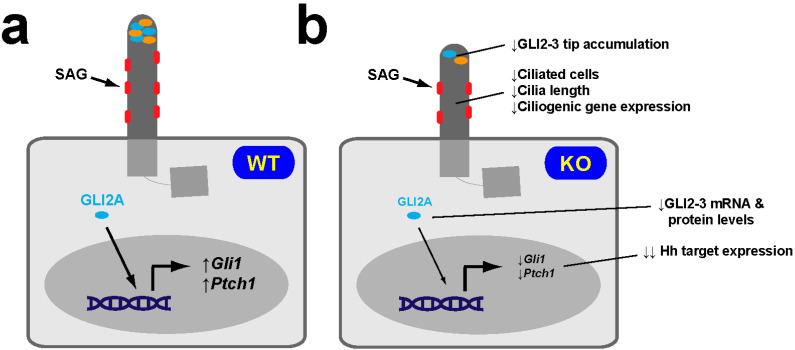
NRF2-null MEFs display reduced SAG-induced Hh responsiveness. (**a**) Hh pathway activation by the SMO agonist SAG in WT MEFs causes robust accumulation at the ciliary tip of GLI2 and GLI3 (both encoded by genes that contain canonical ARE sequences in their promoters), and robust induction of the Hh target genes *Gli1* and *Ptch1*. (**b**) SAG-treated NRF2-KO MEFs express *Gli1* and *Ptch1* at much lower levels, which is consistent with these cells having fewer and shorter cilia, lower expression levels of ciliogenic genes, reduced *Gli2* and *Gli3* mRNA levels, and reduced overall levels and ciliary tip accumulation of GLI2 and GLI3 proteins. SMO, GLI2 and GLI3 are shown in red, cyan and orange, respectively [58].

Taken together, these data indicate that NRF2 promotes ciliogenesis and Hh signaling by stimulating, in some cases directly, the expression of genes associated with both these processes (Figure 5) [58].

However, recent work indicates that NRF2 can also function as a negative regulator of ciliogenesis and Hh pathway activity [59]. It is therefore of interest to review these data and discuss whether and how NRF2 may act both positively and negatively in these processes.

## 5. NRF2 Downregulation of Primary Cilia and Hh Signaling

Although NRF2 is cytoprotective under normal conditions, excessive NRF2 activity is oncogenic [110,111,112,113,114,115,116,117,118,119,120]. For instance, NRF2 is frequently upregulated in non-small cell lung carcinoma (NSCLC), where it stimulates tumorigenesis by activating key serine-glycine biosynthetic genes, thereby providing precursors for glutathione and nucleotide production [111,120]. However, other mechanisms may also mediate NRF2′s effect on NSCLC progression. In a recent study, Liu et al. provide evidence that NRF2-mediated downregulation of ciliogenesis and Hh signaling may also help explain the oncogenic effects of NRF2 [59]. In the following sections we review these findings and suggest possible explanations wherever these new data appear to contradict our own [58,59].

### 5.1. NRF2 Downregulation of Ciliogenesis

Using MEFs and three NSCLC cell lines (BEAS-2B, H838 and H1299), Liu et al. show that NRF2-null cells have increased ciliogenesis, whereas NRF2 overexpression or stabilization (by pharmacological or genetic inactivation of KEAP1) reduces ciliation. Moreover, NRF2 is shown to repress the expression of ciliogenic genes and proteins (*KIF3A*, *IFT88* and *IFT20*), and to reduce the levels of two ciliary proteins, ARL13B and acetylated tubulin (AcTub) [59]. Interestingly, Liu et al. also find that ciliobrevin (HPI-4), a compound known to repress ciliogenesis, is in fact an NRF2 inducer acting canonically via KEAP1 Cys-151, as do electrophiles like dimethyl fumarate and the apocarotenoid bixin [59,106,107,121].

How can these data be reconciled with our findings that NRF2 promotes ciliogenesis and ciliogenic gene expression [58,59]? Our best guess is that the effects of NRF2 are context-dependent. Since we studied MEFs and mouse hippocampus, the different behavior of NRF2 in NSCLC cell lines may be due to cell type-specific differences in NRF2 regulation or target gene accessibility. Harder to explain is why NRF2 would promote ciliogenesis in one set of MEF experiments while inhibiting it in the other. Nevertheless, even here, context may be key, as not all MEFs are created equal [122]. Indeed, different MEF sets not only come from different mouse litters but several other factors may distinguish them as well: (i) mouse colonies are kept in different labs under different environments; (ii) mouse strains of origin can have different genetic backgrounds; (iii) the embryonic age of MEF derivation may differ; (iv) there may be differences in protocols used for their derivation, propagation and immortalization; (v) different number of passages after derivation; and (vi) stochastic variation between different MEF derivations and genetic drift during passaging [122]. At present, it cannot be ruled out that one or more of these factors could account for the observed differences in ciliogenesis. For instance, our MEFs came from the C57BL6 strain, were derived at E11.5 and were immortalized with SV40 large T antigen (as mentioned, our findings were also confirmed in primary MEFs) [58]. No such information is reported by Liu et al., and thus differences in these aspects may well exist [59]. Their MEF culture medium was also different: in addition to DMEM+10% FBS that we also used, theirs contained non-essential amino acids, beta-mercaptoethanol, penicillin and streptomycin [58,59]. Given the complexity of NRF2 regulation and the vast numbers of genes it controls (many of them in a context-dependent manner, as discussed above for *OCT4* and *NANOG*), any of these factors may potentially explain the differences between MEF sets.

Furthermore, since some ciliogenic transcription factors, like RFX1-4, concomitantly regulate multiple ciliogenic genes, all it might take for NRF2 to switch from being a pro-ciliogenic factor to being an anti-ciliogenic one could be to reverse the direction of how it regulates one of these pro-ciliogenic factors [10]. This is actually not such a far-fetched hypothesis: we found that *Rfx4* mRNA was upregulated ≈100-fold in NRF2-null primary MEFs relative to WT, whereas we found no difference in immortalized MEFs, even though ciliogenesis was reduced in both primary and immortalized MEFs. Thus, these data demonstrate that NRF2 regulates RFX4 in a context-dependent manner [58]. Further research is warranted to clarify these issues.

Besides ciliogenic gene repression, Liu et al. propose another mechanism for NRF2-dependent inhibition of ciliogenesis: upregulation by NRF2 of its known target, the p62/SQSTM1 autophagy receptor, leads to increased formation of inclusion bodies, ubiquitin-containing protein aggregates whose assembly depends on p62 [75,76,123,124]. NRF2 stabilization increases protein levels and inclusion body targeting of OFD1, a ciliogenic repressor whose autophagic degradation enables ciliogenesis and ciliary entry of BBS4, a ciliopathy-associated protein [68]. Accordingly, NRF2 upregulation antagonizes ciliogenesis and BBS4 ciliary entry in a p62-dependent manner in H1299 cells. In these cells, genetic deletion of p62 completely abolishes ciliogenesis repression by the NRF2 inducer bixin, indicating that reduced ciliogenic gene expression, if it also occurs in this cell line, is not the cause of NRF2-dependent ciliogenesis inhibition, unless such gene repression is also p62-dependent [59]. More studies are needed to elucidate how NRF2 represses ciliogenesis in these models.

### 5.2. NRF2 Downregulation of Hh Signaling

Besides ciliogenesis, Liu et al. also reported increased Hh signaling in MEFs and NSCLC lines [59]. Here, their data are even less comparable to ours than the data on ciliogenesis. First, our Hh responsiveness assays involved SAG-induced upregulation of endogenous *Gli1* and *Ptch1* [58]. On the other hand, Liu et al. investigated how recombinant SHH stimulates expression of a transiently transfected GLI-activated dual luciferase reporter [59]. Although the concepts may appear similar at first glance, there are actually important differences between the different assays. First, SHH acts via PTCH1, while SAG bypasses PTCH1 to directly activate SMO. This is important, because Liu et al. clearly demonstrate that (as we suggested might be the case) *PTCH1* is a bona fide NRF2 target gene, and that PTCH1 protein levels are reduced in NRF2-null cells [58,59]. Since PTCH1 is the SHH receptor, a SMO repressor and a Hh target gene, reductions in its gene and protein expression levels can affect both assays but in different ways (Figure 2) [12].

Second, in our experience, Hh assays combining transient transfection with serum starvation (required for ciliogenesis, without which there is no SHH/SAG responsiveness) tend to be problematic, because this combination is stressful for cells, most of which fail to form cilia under these conditions, leading to poor Hh responsiveness. Accordingly, while our approach, which does not involve transfection, led to 10–15-fold inductions in control cells, the transient reporter assays led at best to 2-fold inductions [58,59]. In fact, in terms of SHH responsiveness, WT and NRF2-null cells (MEFs, H838 and BEAS-2B) behaved almost identically in the latter assays, their main difference being a ≈50% increase in unstimulated reporter activity in the NRF2-null cell lines, consistent with their lower PTCH1 levels. Other than these experiments showing increased basal reporter activity but no clear differences in SHH responses, Liu et al. did not provide further evidence that ligand-induced Hh signaling is affected by NRF2 [59].

**Figure 6:**
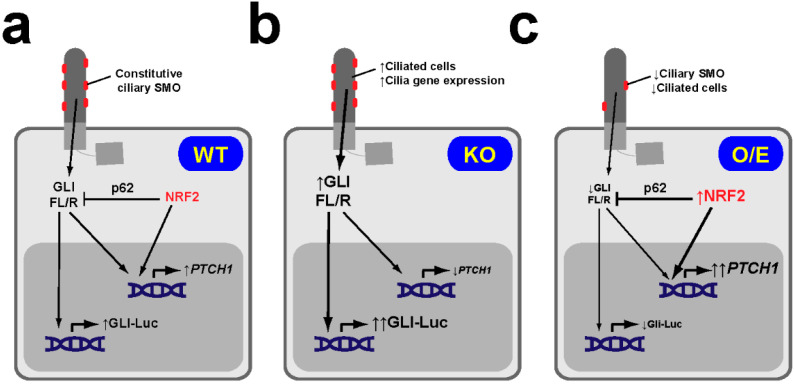
NRF2 downregulates basal Hh pathway activity in NSCLC cells. (**a**) In NRF2 WT NSCLC cell lines like H1299, SMO localizes constitutively at cilia, indicative of active Hh signaling. In these cells, NRF2 levels are low, as are its effects on both *PTCH1* gene expression (which it activates via an ARE in its promoter) and GLI factors (whose full length-to-repressor ratios (FL/R) and transcriptional activity it downregulates via p62/SQSTM1). (**b**) In NRF2-null (KO) H1299 cells, lack of NRF2 leads to increased ciliogenic gene expression and increased ciliogenesis, lower *PTCH1* levels, higher GLI FL/R ratios and higher GLI-luciferase reporter (Gli-Luc) activity. (**c**) In H1299 cells overexpressing (O/E) NRF2 (or where NRF2 is stabilized by pharmacological or genetic KEAP1 inactivation), ciliogenesis is reduced and *PTCH1* is increased, leading to reduced ciliary SMO. Under these conditions, GLI ratios and activity are lower, due to the combined effects of NRF2 on both PTCH1 and p62. Given that *PTCH1* is a transcriptional target of both GLI and NRF2, higher *PTCH1* levels in these cells suggest that NRF2 activation has a stronger effect on *PTCH1* than GLI activity reduction. Similarly, in NRF2 KO H1299 cells (**b**), NRF2 loss predominates over increased GLI activity [59].

On the other hand, Liu et al. provide good evidence that NRF2 negatively regulates basal GLI reporter activity, especially in the H1299 NSCLC line (Figure 6). In these cells, NRF2 overexpression or bixin-induced stabilization reduces GLI-luciferase reporter activity by 30%–40%. Likewise, the ciliary localization of SMO is reduced 2-fold by these manipulations or by KEAP1 genetic deletion. These data show that SMO is constitutively at cilia in H1299 cells, indicating they have a constitutively active Hh pathway [59]. Since Hh signaling is well-known to play a major role in NSCLC progression, this suggests that the negative effects of NRF2 on this pathway may antagonize tumorigenicity rather than promote it, as suggested by Liu et al. [59,125,126,127,128,129,130,131]. This, however, remains untested.

The other main assay that Liu et al. employed for Hh pathway analysis was an immunoblot assay for GLI2 and GLI3, with quantitation of full-length-to-repressor ratios (GLI2FL/GLI2R and GLI3FL/GLI3R). Although these assays provide valuable information to nicely complement the GLI transcriptional activity assays, using these ratios as the main method to assess Hh pathway activity is not without caveats. Regarding GLI2, its function relies mostly on its transformation from GLI2FL to GLI2A, the activator whose SDS-PAGE migration is indistinguishable from that of GLI2FL. Additionally, as previously shown (and as immunoblots by Liu et al. repeatedly confirm), GLI2FL processing into GLI2R is inefficient, which means GLI2R levels are low and thus its role in the pathway likely minor (Figure 2) [16,132,133]. Hence, the GLI2FL/GLI2R ratio may not correlate well with Hh pathway activation: even if such a correlation is expected in WT cells, it has not been properly demonstrated that Hh target gene activity and the GLI2FL/GLI2R ratio also correlate well in NRF2-null cells.

The GLI3FL/GLI3R ratio is a better measure, because GLI3R plays a major and direct role in repressing Hh target gene transcription. While WT cells show a clear increase in this ratio in response to Hh ligands, it is again not clear whether this correlation holds true also in NRF2-null cells, where GLI3R activity may be affected by changes (phosphorylation, nuclear translocation, etc.) that may not necessarily be reflected in an altered GLI3FL/GLI3R ratio [16,132,133]. In other words, while Hh ligands are known to increase the GLI3FL/GLI3R ratio by blocking GLI3FL-to-GLI3R processing, the mechanism whereby NRF2 alters this ratio is still unknown. In fact, the data by Liu et al. suggest mechanistic differences, as discussed next.

Thus, if individual GLI3FL and GLI3R bands are compared to a loading control (e.g., GAPDH), Hh ligands often affect the GLI3FL/GAPDH ratio only mildly, while they sharply reduce the GLI3R/GAPDH ratio, thereby increasing the GLI3FL/GLI3R ratio [134,135,136]. In contrast, blots of NRF2-null cells in the study by Liu et al. show increased GLI3FL/GAPDH ratios and virtually unaffected GLI3R/GAPDH ratios. Likewise, NRF2 overexpression or stabilization consistently reduces the GLI3FL/GAPDH ratio while barely affecting the GLI3R/GAPDH ratio [59]. Although no Hh ligand-treated controls appear in these blots for comparison, these effects of NRF2 are very consistent and they also apply to GLI2. Effects on GLI2R and GLI3R, when discernible, are only seen long after the changes in GLI2FL and GLI3FL, which suggests that the former changes are an indirect consequence of the latter ones [59].

Since these effects of NRF2 are abolished in p62 KO cells [59], we hypothesize that NRF2 negatively controls GLI2FL and GLI3FL protein levels in a p62-dependent manner. Possible mediators of these effects include the Hh pathway repressor SUFU, which specifically stabilizes GLI2FL and GLI3FL without affecting GLI2R and GLI3R (and whose gene expression we showed to be NRF2-dependent), and SPOP, a Cullin-3 ubiquitin ligase adaptor that targets GLI2FL and GLI3FL (but not GLI2R and GLI3R) for degradation [16,58,135,137]. These hypotheses await empirical testing.

Liu et al. also address the mechanisms of NRF2-dependent downregulation of basal Hh pathway activity [59]. They show convincingly that NRF2 directly activates *PTCH1* gene expression by specifically binding to an ARE in its promoter, thereby leading to higher PTCH1 protein levels, not only in cell lines but also in NRF2-overexpressing lung tumors. Increased PTCH1 in turn reduces the amount of ciliary SMO, a reduction that is fully abolished in PTCH1-null H1299 cells. In these KO cells, however, bixin stabilization of NRF2 still significantly reduces GLI reporter activity, as it does in WT cells (≈40% reduction in WT versus ≈25% reduction in PTCH1-null cells; not significantly different). Interestingly, while p62-null H1299 cells behave almost identically to PTCH1-null cells (bixin reduces GLI reporter activity ≈25% versus ≈45% in control; not significantly different), bixin has no activity when p62 is knocked down in PTCH1-null cells. Taken together, these findings suggest that the NRF2-dependent reduction in GLI activity results from additive effects mediated by both PTCH1 and p62. While PTCH1 is a known pathway repressor, the repressive p62 effects presumably result from its above-stated effects on both ciliogenesis and GLI proteins. Therefore, although many molecular details remain to be elucidated, Liu et al. provide credible explanations regarding how NRF2 represses ciliogenesis and Hh signaling in their models [59].

## 6. Concluding Remarks

Only five years ago, the fields of NRF2 and primary cilia were completely independent from each other. The discovery of the PAN axis in hESCs demonstrated that primary cilia signal to NRF2 [57], thereby prompting other groups (including ourselves) to look for additional connections between NRF2 and cilia. This led to the realization that NRF2 also affects primary cilia and their signaling functions. Not only is the NRF2-cilia relationship bidirectional, but the effects of NRF2 on cilia also seem to be switchable between positive and negative depending on contextual factors that are still poorly understood. Our understanding of the NRF2-cilia interplay is thus still incomplete and many exciting discoveries likely lie ahead [57,58,59].

A major unanswered question is the pathophysiological significance of the NRF2-cilia connections. This has been addressed only for the PAN axis in hESCs [57]. In Section 3, we already speculated on how the PAN axis might determine other stem cell fates, or how it might allow differentiated cells to sense ciliary stress or damage. A related possibility is that cilia sense extracellular molecules whose actions trigger intracellular stress (e.g., cilia might sense xenobiotics before they enter cells and cause redox stress). These stress-purveying molecules might act by inactivating the PAN axis, thus leading to higher NRF2 activity and stronger defenses against redox and other stresses. Further studies are needed to address these hypotheses.

Even less clear is the biological meaning of the roles of NRF2 in ciliogenesis and Hh signaling. NRF2-null mice are viable (even if more susceptible to stress than WT mice), while mice lacking important ciliary or Hh components die in utero and display abundant malformations. Thus, it is clear that the connections between NRF2 and cilia (including the PAN axis) are of little consequence during normal embryo development, no matter how important they may prove to be under stress or later in life [12,52,53,138,139,140]. This raises several questions, such as whether other factors are redundant with NRF2 during development, or where and when the signaling between NRF2 and cilia is important for health and disease. An interesting possibility is that ciliogenesis and Hh signaling may also contribute to the cytoprotective functions of NRF2. Consistent with this, several reports indicate that redox stress affects primary cilia and Hh signaling, which in turn protect cells against such stress [39,40,41,42,43,44,45,46,47,48,49,50,51].

Our study, where we discussed these issues at length, showed that astrocyte cilia are strongly affected in the hippocampus of adult NRF2-null mice, even though ependymal motile cilia lining their brain ventricles look normal. Thus, hippocampal function may be affected by NRF2-dependent effects on cilia [58,87,88,89,90,91,92,93,94,95,96,97,98]. On the other hand, Liu et al. showed that the NRF2-cilia axis may be important in NSCLC and other cancers [59,111,114,120,125]. Now that the NRF2-primary cilia partnership has been discovered, it is only a matter of time until its biological functions are elucidated.

## Figures and Tables

**Table 1:** Primary cilia and human diseases.

Tissue/Cell Type	Ciliary Signal	Manifestation	Diseases ^1^
Retinal photoreceptors	Photons	Retinal degeneration	RP, LCA, BBS
Kidney tubules	Urine flow	Cystic kidneys	PKD, NPHP, MKS
Hypothalamic neurons	Neuropeptides	Hyperphagia	Obesity, BBS
Olfactory neurons	Odorants	Anosmia	BBS
Embryonic neural tube	SHH	Brain malformations	MKS, JBTS
Embryonic limb bud	SHH	Polydactyly	MKS, JBTS, BBS, EVC
Chondrocytes	IHH	Skeletal dysplasia	JATD, EVC
Granule neuron progenitors	SHH	Cerebellar cancer	Medulloblastoma
Epidermal stem cells	SHH	Skin cancer	Basal cell carcinoma

^1^ Diseases mentioned are examples (e.g., cystic kidneys or skeletal dysplasia are also found in other ciliopathies). Acronyms: retinitis pigmentosa (RP); Leber congenital amaurosis (LCA); Bardet–Biedl syndrome (BBS); polycystic kidney disease (PKD); nephronophthisis (NPHP); Meckel–Gruber syndrome (MKS); Joubert–Boltshauser syndrome (JBTS); Ellis-van Creveld syndrome (EVC); Jeune asphyxiating thoracic dystrophy (JATD) [14,21,23].

## References

[1] Reiter J.F., Leroux M.R. (2017). Genes and molecular pathways underpinning ciliopathies. Nat. Rev. Mol. Cell Biol..

[2] Anvarian Z., Mykytyn K., Mukhopadhyay S., Pedersen L.B., Christensen S.T. (2019). Cellular signalling by primary cilia in development, organ function and disease. Nat. Rev. Nephrol..

[3] Nachury M.V., Mick D.U. (2019). Establishing and regulating the composition of cilia for signal transduction. Nat. Rev. Mol. Cell Biol..

[4] Garcia-Gonzalo F.R., Reiter J.F. (2017). Open Sesame: How Transition Fibers and the Transition Zone Control Ciliary Composition. Cold Spring Harb. Perspect. Biol..

[5] Garcia-Gonzalo F.R., Reiter J.F. (2012). Scoring a backstage pass: Mechanisms of ciliogenesis and ciliary access. J. Cell Biol..

[6] Wang L., Dynlacht B.D. (2018). The regulation of cilium assembly and disassembly in development and disease. Development.

[7] Cassioli C., Baldari C.T. (2019). A Ciliary View of the Immunological Synapse. Cells.

[8] Ishikawa H., Marshall W.F. (2017). Intraflagellar Transport and Ciliary Dynamics. Cold Spring Harb. Perspect. Biol..

[9] Taschner M., Lorentzen E. (2016). The Intraflagellar Transport Machinery. Cold Spring Harb. Perspect. Biol..

[10] Choksi S.P., Lauter G., Swoboda P., Roy S. (2014). Switching on cilia: Transcriptional networks regulating ciliogenesis. Development.

[11] Neugebauer J.M., Amack J.D., Peterson A.G., Bisgrove B.W., Yost H.J. (2009). FGF signalling during embryo development regulates cilia length in diverse epithelia. Nature.

[12] Bangs F., Anderson K.V. (2017). Primary Cilia and Mammalian Hedgehog Signaling. Cold Spring Harb. Perspect. Biol..

[13] Raleigh D.R., Reiter J.F. (2019). Misactivation of Hedgehog signaling causes inherited and sporadic cancers. J. Clin. Investig..

[14] Eguether T., Hahne M. (2018). Mixed signals from the cell’s antennae: Primary cilia in cancer. EMBO Rep..

[15] Briscoe J., Therond P.P. (2013). The mechanisms of Hedgehog signalling and its roles in development and disease. Nat. Rev. Mol. Cell Biol..

[16] Niewiadomski P., Niedziolka S.M., Markiewicz L., Uspienski T., Baran B., Chojnowska K. (2019). Gli Proteins: Regulation in Development and Cancer. Cells.

[17] Mykytyn K., Askwith C. (2017). G-Protein-Coupled Receptor Signaling in Cilia. Cold Spring Harb. Perspect. Biol..

[18] Chaumont-Dubel S., Dupuy V., Bockaert J., Becamel C., Marin P. (2019). The 5-HT6 receptor interactome: New insight in receptor signaling and its impact on brain physiology and pathologies. Neuropharmacology.

[19] Armato U., Chakravarthy B., Pacchiana R., Whitfield J.F. (2013). Alzheimer’s disease: An update of the roles of receptors, astrocytes and primary cilia (review). Int. J. Mol. Med..

[20] Bergmann C., Guay-Woodford L.M., Harris P.C., Horie S., Peters D.J.M., Torres V.E. (2018). Polycystic kidney disease. Nat. Rev. Dis. Prim..

[21] Braun D.A., Hildebrandt F. (2017). Ciliopathies. Cold Spring Harb. Perspect. Biol..

[22] Siljee J.E., Wang Y., Bernard A.A., Ersoy B.A., Zhang S., Marley A., Von Zastrow M., Reiter J.F., Vaisse C. (2018). Subcellular localization of MC4R with ADCY3 at neuronal primary cilia underlies a common pathway for genetic predisposition to obesity. Nat. Genet..

[23] Vaisse C., Reiter J.F., Berbari N.F. (2017). Cilia and Obesity. Cold Spring Harb. Perspect. Biol..

[24] Dhekne H.S., Yanatori I., Gomez R.C., Tonelli F., Diez F., Schule B., Steger M., Alessi D.R., Pfeffer S.R. (2018). A pathway for Parkinson’s Disease LRRK2 kinase to block primary cilia and Sonic hedgehog signaling in the brain. Elife.

[25] Hu L., Wang B., Zhang Y. (2017). Serotonin 5-HT6 receptors affect cognition in a mouse model of Alzheimer’s disease by regulating cilia function. Alzheimers Res. Ther..

[26] Kaliszewski M., Knott A.B., Bossy-Wetzel E. (2015). Primary cilia and autophagic dysfunction in Huntington’s disease. Cell Death Differ..

[27] Kluth O., Stadion M., Gottmann P., Aga H., Jahnert M., Scherneck S., Vogel H., Krus U., Seelig A., Ling C. (2019). Decreased Expression of Cilia Genes in Pancreatic Islets as a Risk Factor for Type 2 Diabetes in Mice and Humans. Cell Rep..

[28] Volta F., Gerdes J.M. (2017). The role of primary cilia in obesity and diabetes. Ann. N. Y. Acad. Sci..

[29] Kopinke D., Roberson E.C., Reiter J.F. (2017). Ciliary Hedgehog Signaling Restricts Injury-Induced Adipogenesis. Cell.

[30] Hilgendorf K.I., Johnson C.T., Mezger A., Rice S.L., Norris A.M., Demeter J., Greenleaf W.J., Reiter J.F., Kopinke D., Jackson P.K. (2019). Omega-3 Fatty Acids Activate Ciliary FFAR4 to Control Adipogenesis. Cell.

[31] Cheignon C., Tomas M., Bonnefont-Rousselot D., Faller P., Hureau C., Collin F. (2018). Oxidative stress and the amyloid beta peptide in Alzheimer’s disease. Redox Biol..

[32] Kirtonia A., Sethi G., Garg M. (2020). The multifaceted role of reactive oxygen species in tumorigenesis. Cell. Mol. Life Sci..

[33] McMurray F., Patten D.A., Harper M.E. (2016). Reactive Oxygen Species and Oxidative Stress in Obesity-Recent Findings and Empirical Approaches. Obesity.

[34] Petrillo S., Pelosi L., Piemonte F., Travaglini L., Forcina L., Catteruccia M., Petrini S., Verardo M., D’Amico A., Musaro A. (2017). Oxidative stress in Duchenne muscular dystrophy: Focus on the NRF2 redox pathway. Hum. Mol. Genet..

[35] Poprac P., Jomova K., Simunkova M., Kollar V., Rhodes C.J., Valko M. (2017). Targeting Free Radicals in Oxidative Stress-Related Human Diseases. Trends Pharmacol. Sci..

[36] Rochette L., Zeller M., Cottin Y., Vergely C. (2018). Redox Functions of Heme Oxygenase-1 and Biliverdin Reductase in Diabetes. Trends Endocrinol. Metab..

[37] Rossetti A.C., Paladini M.S., Riva M.A., Molteni R. (2020). Oxidation-reduction mechanisms in psychiatric disorders: A novel target for pharmacological intervention. Pharmacol. Ther..

[38] van Dam L., Dansen T.B. (2020). Cross-talk between redox signalling and protein aggregation. Biochem. Soc. Trans..

[39] Chen K.Y., Chiu C.H., Wang L.C. (2017). Anti-apoptotic effects of Sonic hedgehog signalling through oxidative stress reduction in astrocytes co-cultured with excretory-secretory products of larval Angiostrongylus cantonensis. Sci. Rep..

[40] Dai R.L., Zhu S.Y., Xia Y.P., Mao L., Mei Y.W., Yao Y.F., Xue Y.M., Hu B. (2011). Sonic hedgehog protects cortical neurons against oxidative stress. Neurochem. Res..

[41] Das S., Jackson W.P., Prasain J.K., Hanna A., Bailey S.K., Tucker J.A., Bae S., Wilson L.S., Samant R.S., Barnes S. (2017). Loss of Merlin induces metabolomic adaptation that engages dependence on Hedgehog signaling. Sci. Rep..

[42] Hai B., Zhao Q., Deveau M.A., Liu F. (2018). Delivery of Sonic Hedgehog Gene Repressed Irradiation-induced Cellular Senescence in Salivary Glands by Promoting DNA Repair and Reducing Oxidative Stress. Theranostics.

[43] He W., Cui L., Zhang C., Zhang X., He J., Xie Y., Chen Y. (2017). Sonic hedgehog promotes neurite outgrowth of cortical neurons under oxidative stress: Involving of mitochondria and energy metabolism. Exp. Cell Res..

[44] Kaushal J.B., Popli P., Sankhwar P., Shukla V., Dwivedi A. (2018). Sonic hedgehog protects endometrial hyperplasial cells against oxidative stress via suppressing mitochondrial fission protein dynamin-like GTPase (Drp1). Free. Radic. Biol. Med..

[45] Kim W.K., Meliton V., Bourquard N., Hahn T.J., Parhami F. (2010). Hedgehog signaling and osteogenic differentiation in multipotent bone marrow stromal cells are inhibited by oxidative stress. J. Cell. Biochem..

[46] Lin E.H., Kao Y.R., Lin C.A., Kuo T.Y., Yang S.P., Hsu C.F., Chou T.Y., Ho C.C., Wu C.W. (2016). Hedgehog pathway maintains cell survival under stress conditions, and drives drug resistance in lung adenocarcinoma. Oncotarget.

[47] Bae J.E., Kang G.M., Min S.H., Jo D.S., Jung Y.K., Kim K., Kim M.S., Cho D.H. (2019). Primary cilia mediate mitochondrial stress responses to promote dopamine neuron survival in a Parkinson’s disease model. Cell Death Dis..

[48] Han S.J., Jang H.S., Seu S.Y., Cho H.J., Hwang Y.J., Kim J.I., Park K.M. (2017). Hepatic ischemia/reperfusion injury disrupts the homeostasis of kidney primary cilia via oxidative stress. Biochim. Biophys. Acta Mol. Basis Dis..

[49] Kim J.I., Kim J., Jang H.S., Noh M.R., Lipschutz J.H., Park K.M. (2013). Reduction of oxidative stress during recovery accelerates normalization of primary cilia length that is altered after ischemic injury in murine kidneys. Am. J. Physiol. Renal. Physiol..

[50] Lavagnino M., Oslapas A.N., Gardner K.L., Arnoczky S.P. (2016). Hypoxia inhibits primary cilia formation and reduces cell-mediated contraction in stress-deprived rat tail tendon fascicles. Muscles Ligaments Tendons J..

[51] Siroky B.J., Kleene N.K., Kleene S.J., Varnell C.D., Comer R.G., Liu J., Lu L., Pachciarz N.W., Bissler J.J., Dixon B.P. (2017). Primary cilia regulate the osmotic stress response of renal epithelial cells through TRPM3. Am. J. Physiol. Renal. Physiol..

[52] Cuadrado A., Manda G., Hassan A., Alcaraz M.J., Barbas C., Daiber A., Ghezzi P., Leon R., Lopez M.G., Oliva B. (2018). Transcription Factor NRF2 as a Therapeutic Target for Chronic Diseases: A Systems Medicine Approach. Pharmacol. Rev..

[53] Tonelli C., Chio I.I.C., Tuveson D.A. (2018). Transcriptional Regulation by Nrf2. Antioxid Redox Signal..

[54] Cuadrado A., Rojo A.I., Wells G., Hayes J.D., Cousin S.P., Rumsey W.L., Attucks O.C., Franklin S., Levonen A.L., Kensler T.W. (2019). Therapeutic targeting of the NRF2 and KEAP1 partnership in chronic diseases. Nat. Rev. Drug Discov..

[55] Pajares M., Rojo A.I., Arias E., Diaz-Carretero A., Cuervo A.M., Cuadrado A. (2018). Transcription factor NFE2L2/NRF2 modulates chaperone-mediated autophagy through the regulation of LAMP2A. Autophagy.

[56] Pajares M., Jimenez-Moreno N., Garcia-Yague A.J., Escoll M., de Ceballos M.L., Van Leuven F., Rabano A., Yamamoto M., Rojo A.I., Cuadrado A. (2016). Transcription factor NFE2L2/NRF2 is a regulator of macroautophagy genes. Autophagy.

[57] Jang J., Wang Y., Lalli M.A., Guzman E., Godshalk S.E., Zhou H., Kosik K.S. (2016). Primary Cilium-Autophagy-Nrf2 (PAN) Axis Activation Commits Human Embryonic Stem Cells to a Neuroectoderm Fate. Cell.

[58] Martin-Hurtado A., Martin-Morales R., Robledinos-Anton N., Blanco R., Palacios-Blanco I., Lastres-Becker I., Cuadrado A., Garcia-Gonzalo F.R. (2019). NRF2-dependent gene expression promotes ciliogenesis and Hedgehog signaling. Sci. Rep..

[59] Liu P., Dodson M., Fang D., Chapman E., Zhang D.D. (2020). NRF2 negatively regulates primary ciliogenesis and hedgehog signaling. PLoS Biol..

[60] Levine B., Kroemer G. (2019). Biological Functions of Autophagy Genes: A Disease Perspective. Cell.

[61] Lopez-Otin C., Kroemer G. (2019). Decelerating ageing and biological clocks by autophagy. Nat. Rev. Mol. Cell Biol..

[62] Dikic I., Elazar Z. (2018). Mechanism and medical implications of mammalian autophagy. Nat. Rev. Mol. Cell Biol..

[63] Hansen M., Rubinsztein D.C., Walker D.W. (2018). Autophagy as a promoter of longevity: Insights from model organisms. Nat. Rev. Mol. Cell Biol..

[64] Morleo M., Franco B. (2019). The Autophagy-Cilia Axis: An Intricate Relationship. Cells.

[65] Pampliega O., Cuervo A.M. (2016). Autophagy and primary cilia: Dual interplay. Curr. Opin. Cell Biol..

[66] Liu Z.Q., Lee J.N., Son M., Lim J.Y., Dutta R.K., Maharjan Y., Kwak S., Oh G.T., Byun K., Choe S.K. (2018). Ciliogenesis is reciprocally regulated by PPARA and NR1H4/FXR through controlling autophagy in vitro and in vivo. Autophagy.

[67] Pampliega O., Orhon I., Patel B., Sridhar S., Diaz-Carretero A., Beau I., Codogno P., Satir B.H., Satir P., Cuervo A.M. (2013). Functional interaction between autophagy and ciliogenesis. Nature.

[68] Tang Z., Lin M.G., Stowe T.R., Chen S., Zhu M., Stearns T., Franco B., Zhong Q. (2013). Autophagy promotes primary ciliogenesis by removing OFD1 from centriolar satellites. Nature.

[69] Wang S., Livingston M.J., Su Y., Dong Z. (2015). Reciprocal regulation of cilia and autophagy via the MTOR and proteasome pathways. Autophagy.

[70] Jimenez-Sanchez M., Menzies F.M., Chang Y.Y., Simecek N., Neufeld T.P., Rubinsztein D.C. (2012). The Hedgehog signalling pathway regulates autophagy. Nat. Commun..

[71] Ichimura Y., Waguri S., Sou Y.S., Kageyama S., Hasegawa J., Ishimura R., Saito T., Yang Y., Kouno T., Fukutomi T. (2013). Phosphorylation of p62 activates the Keap1-Nrf2 pathway during selective autophagy. Mol. Cell..

[72] Jena K.K., Kolapalli S.P., Mehto S., Nath P., Das B., Sahoo P.K., Ahad A., Syed G.H., Raghav S.K., Senapati S. (2018). TRIM16 controls assembly and degradation of protein aggregates by modulating the p62-NRF2 axis and autophagy. EMBO J..

[73] Komatsu M., Kurokawa H., Waguri S., Taguchi K., Kobayashi A., Ichimura Y., Sou Y.S., Ueno I., Sakamoto A., Tong K.I. (2010). The selective autophagy substrate p62 activates the stress responsive transcription factor Nrf2 through inactivation of Keap1. Nat. Cell. Biol..

[74] Lau A., Wang X.J., Zhao F., Villeneuve N.F., Wu T., Jiang T., Sun Z., White E., Zhang D.D. (2010). A noncanonical mechanism of Nrf2 activation by autophagy deficiency: Direct interaction between Keap1 and p62. Mol. Cell. Biol..

[75] Fujita K., Maeda D., Xiao Q., Srinivasula S.M. (2011). Nrf2-mediated induction of p62 controls Toll-like receptor-4-driven aggresome-like induced structure formation and autophagic degradation. Proc. Natl. Acad. Sci. USA.

[76] Jain A., Lamark T., Sjottem E., Larsen K.B., Awuh J.A., Overvatn A., McMahon M., Hayes J.D., Johansen T. (2010). p62/SQSTM1 is a target gene for transcription factor NRF2 and creates a positive feedback loop by inducing antioxidant response element-driven gene transcription. J. Biol. Chem..

[77] Lam H.C., Cloonan S.M., Bhashyam A.R., Haspel J.A., Singh A., Sathirapongsasuti J.F., Cervo M., Yao H., Chung A.L., Mizumura K. (2013). Histone deacetylase 6-mediated selective autophagy regulates COPD-associated cilia dysfunction. J. Clin. Investig..

[78] Boehlke C., Kotsis F., Patel V., Braeg S., Voelker H., Bredt S., Beyer T., Janusch H., Hamann C., Godel M. (2010). Primary cilia regulate mTORC1 activity and cell size through Lkb1. Nat. Cell. Biol..

[79] Orhon I., Dupont N., Zaidan M., Boitez V., Burtin M., Schmitt A., Capiod T., Viau A., Beau I., Kuehn E.W. (2016). Primary-cilium-dependent autophagy controls epithelial cell volume in response to fluid flow. Nat. Cell. Biol..

[80] Grantham J.J., Geiser J.L., Evan A.P. (1987). Cyst formation and growth in autosomal dominant polycystic kidney disease. Kidney Int..

[81] Adam J., Hatipoglu E., O’Flaherty L., Ternette N., Sahgal N., Lockstone H., Baban D., Nye E., Stamp G.W., Wolhuter K. (2011). Renal cyst formation in Fh1-deficient mice is independent of the Hif/Phd pathway: Roles for fumarate in KEAP1 succination and Nrf2 signaling. Cancer Cell..

[82] Raleigh D.R., Sever N., Choksi P.K., Sigg M.A., Hines K.M., Thompson B.M., Elnatan D., Jaishankar P., Bisignano P., Garcia-Gonzalo F.R. (2018). Cilia-Associated Oxysterols Activate Smoothened. Mol. Cell..

[83] Garcia-Gonzalo F.R., Phua S.C., Roberson E.C., Garcia G., Abedin M., Schurmans S., Inoue T., Reiter J.F. (2015). Phosphoinositides Regulate Ciliary Protein Trafficking to Modulate Hedgehog Signaling. Dev. Cell..

[84] Yee L.E., Garcia-Gonzalo F.R., Bowie R.V., Li C., Kennedy J.K., Ashrafi K., Blacque O.E., Leroux M.R., Reiter J.F. (2015). Conserved Genetic Interactions between Ciliopathy Complexes Cooperatively Support Ciliogenesis and Ciliary Signaling. PLoS Genet..

[85] Sang L., Miller J.J., Corbit K.C., Giles R.H., Brauer M.J., Otto E.A., Baye L.M., Wen X., Scales S.J., Kwong M. (2011). Mapping the NPHP-JBTS-MKS protein network reveals ciliopathy disease genes and pathways. Cell.

[86] Chen J.K., Taipale J., Young K.E., Maiti T., Beachy P.A. (2002). Small molecule modulation of Smoothened activity. Proc. Natl. Acad. Sci. USA.

[87] Amador-Arjona A., Elliott J., Miller A., Ginbey A., Pazour G.J., Enikolopov G., Roberts A.J., Terskikh A.V. (2011). Primary cilia regulate proliferation of amplifying progenitors in adult hippocampus: Implications for learning and memory. J. Neurosci..

[88] Berbari N.F., Malarkey E.B., Yazdi S.M., McNair A.D., Kippe J.M., Croyle M.J., Kraft T.W., Yoder B.K. (2014). Hippocampal and cortical primary cilia are required for aversive memory in mice. PLoS ONE.

[89] Rhee S., Kirschen G.W., Gu Y., Ge S. (2016). Depletion of primary cilia from mature dentate granule cells impairs hippocampus-dependent contextual memory. Sci. Rep..

[90] Yao P.J., Petralia R.S., Mattson M.P. (2016). Sonic Hedgehog Signaling and Hippocampal Neuroplasticity. Trends Neurosci..

[91] Robledinos-Anton N., Rojo A.I., Ferreiro E., Nunez A., Krause K.H., Jaquet V., Cuadrado A. (2017). Transcription factor NRF2 controls the fate of neural stem cells in the subgranular zone of the hippocampus. Redox Biol..

[92] Ray S., Corenblum M.J., Anandhan A., Reed A., Ortiz F.O., Zhang D.D., Barnes C.A., Madhavan L. (2018). A Role for Nrf2 Expression in Defining the Aging of Hippocampal Neural Stem Cells. Cell Transplant..

[93] Hill S.A., Blaeser A.S., Coley A.A., Xie Y., Shepard K.A., Harwell C.C., Gao W.J., Garcia A.D.R. (2019). Sonic hedgehog signaling in astrocytes mediates cell-type-specific synaptic organization. Elife.

[94] Farmer W.T., Abrahamsson T., Chierzi S., Lui C., Zaelzer C., Jones E.V., Bally B.P., Chen G.G., Theroux J.F., Peng J. (2016). Neurons diversify astrocytes in the adult brain through sonic hedgehog signaling. Science.

[95] Liddell J.R. (2017). Are Astrocytes the Predominant Cell Type for Activation of Nrf2 in Aging and Neurodegeneration?. Antioxidants.

[96] Gan L., Vargas M.R., Johnson D.A., Johnson J.A. (2012). Astrocyte-specific overexpression of Nrf2 delays motor pathology and synuclein aggregation throughout the CNS in the alpha-synuclein mutant (A53T) mouse model. J. Neurosci..

[97] Chen P.C., Vargas M.R., Pani A.K., Smeyne R.J., Johnson D.A., Kan Y.W., Johnson J.A. (2009). Nrf2-mediated neuroprotection in the MPTP mouse model of Parkinson’s disease: Critical role for the astrocyte. Proc. Natl. Acad. Sci. USA.

[98] Leung L., Kwong M., Hou S., Lee C., Chan J.Y. (2003). Deficiency of the Nrf1 and Nrf2 transcription factors results in early embryonic lethality and severe oxidative stress. J. Biol. Chem..

[99] Peixoto E., Jin S., Thelen K., Biswas A., Richard S., Morleo M., Mansini A., Holtorf S., Carbone F., Pastore N. (2020). HDAC6-dependent ciliophagy is involved in ciliary loss and cholangiocarcinoma growth in human cells and murine models. Am. J. Physiol. Gastrointest Liver Physiol..

[100] Rushworth G.F., Megson I.L. (2014). Existing and potential therapeutic uses for N-acetylcysteine: The need for conversion to intracellular glutathione for antioxidant benefits. Pharmacol. Ther..

[101] Sugiaman-Trapman D., Vitezic M., Jouhilahti E.M., Mathelier A., Lauter G., Misra S., Daub C.O., Kere J., Swoboda P. (2018). Characterization of the human RFX transcription factor family by regulatory and target gene analysis. BMC Genom..

[102] Castro W., Chelbi S.T., Niogret C., Ramon-Barros C., Welten S.P.M., Osterheld K., Wang H., Rota G., Morgado L., Vivier E. (2018). The transcription factor Rfx7 limits metabolism of NK cells and promotes their maintenance and immunity. Nat. Immunol..

[103] Manojlovic Z., Earwood R., Kato A., Stefanovic B., Kato Y. (2014). RFX7 is required for the formation of cilia in the neural tube. Mech. Dev..

[104] Chorley B.N., Campbell M.R., Wang X., Karaca M., Sambandan D., Bangura F., Xue P., Pi J., Kleeberger S.R., Bell D.A. (2012). Identification of novel NRF2-regulated genes by ChIP-Seq: Influence on retinoid X receptor alpha. Nucleic Acids Res..

[105] Lastres-Becker I., Garcia-Yague A.J., Scannevin R.H., Casarejos M.J., Kugler S., Rabano A., Cuadrado A. (2016). Repurposing the NRF2 Activator Dimethyl Fumarate as Therapy Against Synucleinopathy in Parkinson’s Disease. Antioxid Redox Signal..

[106] Scannevin R.H., Chollate S., Jung M.Y., Shackett M., Patel H., Bista P., Zeng W., Ryan S., Yamamoto M., Lukashev M. (2012). Fumarates promote cytoprotection of central nervous system cells against oxidative stress via the nuclear factor (erythroid-derived 2)-like 2 pathway. J. Pharmacol. Exp. Ther..

[107] Takaya K., Suzuki T., Motohashi H., Onodera K., Satomi S., Kensler T.W., Yamamoto M. (2012). Validation of the multiple sensor mechanism of the Keap1-Nrf2 system. Free. Radic. Biol. Med..

[108] Eguether T., Cordelieres F.P., Pazour G.J. (2018). Intraflagellar transport is deeply integrated in hedgehog signaling. Mol. Biol. Cell..

[109] Mukhopadhyay S., Wen X., Ratti N., Loktev A., Rangell L., Scales S.J., Jackson P.K. (2013). The ciliary G-protein-coupled receptor Gpr161 negatively regulates the Sonic hedgehog pathway via cAMP signaling. Cell.

[110] Chio I.I.C., Jafarnejad S.M., Ponz-Sarvise M., Park Y., Rivera K., Palm W., Wilson J., Sangar V., Hao Y., Ohlund D. (2016). NRF2 Promotes Tumor Maintenance by Modulating mRNA Translation in Pancreatic Cancer. Cell.

[111] DeNicola G.M., Chen P.H., Mullarky E., Sudderth J.A., Hu Z., Wu D., Tang H., Xie Y., Asara J.M., Huffman K.E. (2015). NRF2 regulates serine biosynthesis in non-small cell lung cancer. Nat. Genet..

[112] DeNicola G.M., Karreth F.A., Humpton T.J., Gopinathan A., Wei C., Frese K., Mangal D., Yu K.H., Yeo C.J., Calhoun E.S. (2011). Oncogene-induced Nrf2 transcription promotes ROS detoxification and tumorigenesis. Nature.

[113] Jiang T., Chen N., Zhao F., Wang X.J., Kong B., Zheng W., Zhang D.D. (2010). High levels of Nrf2 determine chemoresistance in type II endometrial cancer. Cancer Res..

[114] Kitamura H., Motohashi H. (2018). NRF2 addiction in cancer cells. Cancer Sci..

[115] Okazaki K., Papagiannakopoulos T., Motohashi H. (2020). Metabolic features of cancer cells in NRF2 addiction status. Biophys. Rev..

[116] Satoh H., Moriguchi T., Saigusa D., Baird L., Yu L., Rokutan H., Igarashi K., Ebina M., Shibata T., Yamamoto M. (2016). NRF2 Intensifies Host Defense Systems to Prevent Lung Carcinogenesis, but After Tumor Initiation Accelerates Malignant Cell Growth. Cancer Res..

[117] Satoh H., Moriguchi T., Takai J., Ebina M., Yamamoto M. (2013). Nrf2 prevents initiation but accelerates progression through the Kras signaling pathway during lung carcinogenesis. Cancer Res..

[118] Wang H., Liu X., Long M., Huang Y., Zhang L., Zhang R., Zheng Y., Liao X., Wang Y., Liao Q. (2016). NRF2 activation by antioxidant antidiabetic agents accelerates tumor metastasis. Sci. Transl. Med..

[119] Yang H., Xiang S., Kazi A., Sebti S.M. (2020). The GTPase KRAS suppresses the p53 tumor suppressor by activating the NRF2-regulated antioxidant defense system in cancer cells. J. Biol. Chem..

[120] Zeng Z., Wang Z.Y., Li Y.K., Ye D.M., Zeng J., Hu J.L., Chen P.F., Xiao J., Zou J., Li Z.H. (2020). Nuclear factor erythroid 2 (NF-E2)-related factor 2 (Nrf2) in non-small cell lung cancer. Life Sci..

[121] Tao S., Park S.L., Rojo de la Vega M., Zhang D.D., Wondrak G.T. (2015). Systemic administration of the apocarotenoid bixin protects skin against solar UV-induced damage through activation of NRF2. Free. Radic. Biol. Med..

[122] Singhal P.K., Sassi S., Lan L., Au P., Halvorsen S.C., Fukumura D., Jain R.K., Seed B. (2016). Mouse embryonic fibroblasts exhibit extensive developmental and phenotypic diversity. Proc. Natl. Acad. Sci. USA.

[123] Turco E., Witt M., Abert C., Bock-Bierbaum T., Su M.Y., Trapannone R., Sztacho M., Danieli A., Shi X., Zaffagnini G. (2019). FIP200 Claw Domain Binding to p62 Promotes Autophagosome Formation at Ubiquitin Condensates. Mol. Cell..

[124] Zaffagnini G., Savova A., Danieli A., Romanov J., Tremel S., Ebner M., Peterbauer T., Sztacho M., Trapannone R., Tarafder A.K. (2018). p62 filaments capture and present ubiquitinated cargos for autophagy. EMBO J..

[125] Giroux Leprieur E., Jablons D.M., He B. (2018). Old Sonic Hedgehog, new tricks: A new paradigm in thoracic malignancies. Oncotarget.

[126] Giroux Leprieur E., Tolani B., Li H., Leguay F., Hoang N.T., Acevedo L.A., Jin J.Q., Tseng H.H., Yue D., Kim I.J. (2017). Membrane-bound full-length Sonic Hedgehog identifies cancer stem cells in human non-small cell lung cancer. Oncotarget.

[127] Li H., Yue D., Jin J.Q., Woodard G.A., Tolani B., Luh T.M., Giroux-Leprieur E., Mo M., Chen Z., Che J. (2016). Gli promotes epithelial-mesenchymal transition in human lung adenocarcinomas. Oncotarget.

[128] Rodriguez-Blanco J., Schilling N.S., Tokhunts R., Giambelli C., Long J., Fei D.L., Singh S., Black K.E., Wang Z., Galimberti F. (2013). The hedgehog processing pathway is required for NSCLC growth and survival. Oncogene.

[129] Shi S., Deng Y.Z., Zhao J.S., Ji X.D., Shi J., Feng Y.X., Li G., Li J.J., Zhu D., Koeffler H.P. (2012). RACK1 promotes non-small-cell lung cancer tumorigenicity through activating sonic hedgehog signaling pathway. J. Biol. Chem..

[130] Yuan Z., Goetz J.A., Singh S., Ogden S.K., Petty W.J., Black C.C., Memoli V.A., Dmitrovsky E., Robbins D.J. (2007). Frequent requirement of hedgehog signaling in non-small cell lung carcinoma. Oncogene.

[131] Della Corte C.M., Bellevicine C., Vicidomini G., Vitagliano D., Malapelle U., Accardo M., Fabozzi A., Fiorelli A., Fasano M., Papaccio F. (2015). SMO Gene Amplification and Activation of the Hedgehog Pathway as Novel Mechanisms of Resistance to Anti-Epidermal Growth Factor Receptor Drugs in Human Lung Cancer. Clin. Cancer Res..

[132] Pan Y., Bai C.B., Joyner A.L., Wang B. (2006). Sonic hedgehog signaling regulates Gli2 transcriptional activity by suppressing its processing and degradation. Mol. Cell. Biol..

[133] Pan Y., Wang B. (2007). A novel protein-processing domain in Gli2 and Gli3 differentially blocks complete protein degradation by the proteasome. J. Biol. Chem..

[134] Wang B., Fallon J.F., Beachy P.A. (2000). Hedgehog-regulated processing of Gli3 produces an anterior/posterior repressor gradient in the developing vertebrate limb. Cell.

[135] Wen X., Lai C.K., Evangelista M., Hongo J.A., de Sauvage F.J., Scales S.J. (2010). Kinetics of hedgehog-dependent full-length Gli3 accumulation in primary cilia and subsequent degradation. Mol. Cell. Biol..

[136] Humke E.W., Dorn K.V., Milenkovic L., Scott M.P., Rohatgi R. (2010). The output of Hedgehog signaling is controlled by the dynamic association between Suppressor of Fused and the Gli proteins. Genes Dev..

[137] Wang C., Pan Y., Wang B. (2010). Suppressor of fused and Spop regulate the stability, processing and function of Gli2 and Gli3 full-length activators but not their repressors. Development.

[138] Chan K., Lu R., Chang J.C., Kan Y.W. (1996). NRF2, a member of the NFE2 family of transcription factors, is not essential for murine erythropoiesis, growth, and development. Proc. Natl. Acad. Sci. USA.

[139] Huangfu D., Liu A., Rakeman A.S., Murcia N.S., Niswander L., Anderson K.V. (2003). Hedgehog signalling in the mouse requires intraflagellar transport proteins. Nature.

[140] Marszalek J.R., Ruiz-Lozano P., Roberts E., Chien K.R., Goldstein L.S. (1999). Situs inversus and embryonic ciliary morphogenesis defects in mouse mutants lacking the KIF3A subunit of kinesin-II. Proc. Natl. Acad. Sci. USA.

